# Information criterion-based clustering with order-restricted candidate profiles in short time-course microarray experiments

**DOI:** 10.1186/1471-2105-10-146

**Published:** 2009-05-15

**Authors:** Tianqing Liu, Nan Lin, Ningzhong Shi, Baoxue Zhang

**Affiliations:** 1 Key Laboratory for Applied Statistics of MOE and School of Mathematics and Statistics, Northeast Normal University, Changchun, PR China; 2 Department of Mathematics, Washington University in St Louis, St Louis, USA

## Abstract

**Background:**

Time-course microarray experiments produce vector gene expression profiles across a series of time points. Clustering genes based on these profiles is important in discovering functional related and co-regulated genes. Early developed clustering algorithms do not take advantage of the ordering in a time-course study, explicit use of which should allow more sensitive detection of genes that display a consistent pattern over time. Peddada *et al*. [1] proposed a clustering algorithm that can incorporate the temporal ordering using order-restricted statistical inference. This algorithm is, however, very time-consuming and hence inapplicable to most microarray experiments that contain a large number of genes. Its computational burden also imposes difficulty to assess the clustering reliability, which is a very important measure when clustering noisy microarray data.

**Results:**

We propose a computationally efficient information criterion-based clustering algorithm, called ORICC, that also takes account of the ordering in time-course microarray experiments by embedding the order-restricted inference into a model selection framework. Genes are assigned to the profile which they best match determined by a newly proposed information criterion for order-restricted inference. In addition, we also developed a bootstrap procedure to assess ORICC's clustering reliability for every gene. Simulation studies show that the ORICC method is robust, always gives better clustering accuracy than Peddada's method and saves hundreds of times computational time. Under some scenarios, its accuracy is also better than some other existing clustering methods for short time-course microarray data, such as STEM [2] and Wang *et al*. [3]. It is also computationally much faster than Wang *et al*. [3].

**Conclusion:**

Our ORICC algorithm, which takes advantage of the temporal ordering in time-course microarray experiments, provides good clustering accuracy and is meanwhile much faster than Peddada's method. Moreover, the clustering reliability for each gene can also be assessed, which is unavailable in Peddada's method. In a real data example, the ORICC algorithm identifies new and interesting genes that previous analyses failed to reveal.

## Background

The development of microarray technology provides a powerful analytical tool for large scale genomic research. Its ability to simultaneously study thousands of genes under a multitude of conditions presents a huge challenge to comprehend and interpret the resulting mass of data. An important application of microarray technology is to study the dynamic patterns of gene expression across a series of time points and find gene clusters within which genes share similar patterns. The premise is that genes sharing similar expression profiles might be functionally related or co-regulated. Therefore, microarray data may provide insights into gene-gene interactions, gene function and pathway identification. Examples of such studies include response to temperature changes and other stress conditions [4], immune response [5], developmental studies [6], and various systems in the cell [7]. Early clustering analysis of microarray data were mostly on static microarray experiments, such as hierarchial clustering [8], the *k*-nearest neighbors method [9,10], and other correlation-based methods [11,12]. These methods are not designed for time-course microarray data and can not effectively utilize the temporal information. Many clustering algorithms for time-course microarray data have been developed afterwards. Most of them view observed temporal gene expression profiles coming from underlying smooth curves and cluster genes based on estimated expression profiles obtained from nonparametric smoothing [2,13-25]. While these algorithms work well for relatively long time series data, they are not appropriate for short time-course microarray data often taken on a small number of sparse time points. Generally, these algorithms tend to overfit the data when the number of time points is small [2]. A few clustering methods were also proposed specifically for short time-course microarray data, such as [1-3,26,27]. Among them, Peddada *et al*. [1] proposed an interesting idea of using order-restricted inference in clustering short time-course microarray data. A set of candidate expression profiles is first defined by inequality constraints among expression levels at different time points, i.e. up and downs. This strategy is less restrictive than those that define profiles via pre-specified expression levels because only the general shape of the profile is needed. Each candidate profile then represents a potential gene cluster. For short time-course data, the total number of candidate profiles is generally not large. Peddada's method then assigns genes to the cluster represented by its best matched candidate profile determined by some order-restricted statistical inference procedure. Two genes fall into the same cluster if their best matched candidate profiles are the same. This profile matching clustering strategy is different from most unsupervised clustering where a representation of a cluster is often calculated only after the cluster is formed. This method requires no smoothing of the expression profiles and was shown to discover more functionally related genes when applied to a breast cancer cell-line data in [28]. However, Peddada's method is computationally very costly when the number of genes is large. According to our experience on a workstation with a 2.30 GHz AMD Athlon(tm) 64 × 2 Dual Core 4400+ processor and a 2.00 GB memory, it took at least 72 hours to run Peddada's method (implemented in Matlab) for the breast cancer cell line data containing about 1900 genes.

In this article, from a different perspective to the order-restricted inference, we propose a new order-restricted information criterion-based clustering (ORICC) algorithm, which is computationally much more efficient than Peddada's method. Our method selects and clusters genes using the ideas of model selection for order-restricted inference, where estimation makes use of inequalities that define the candidate profiles. The first step is to define candidate profiles and express them in terms of inequalities between the expected gene expression levels at various time points. For a given candidate profile, we estimate the mean expression level at different time points of each gene using the order-restricted maximum likelihood [29]. The best fitting profile for a given gene is then selected using an information criterion for order-restricted inference. Due to the simplicity of our algorithm, the analysis of the breast cancer cell-line data in [28] can now be done in a few minutes using our method.

## Results and discussion

### Inequality profiles

Suppose that a time-course microarray experiment includes *T *time points, and at each time point there are *M *arrays each with *G *genes. Denote *y*_*gti *_the expression measurement of gene *g *at time point *t *on the *i*th array. Suppose that the unknown true mean expression level of gene *g *at time *t *is *μ*_*gt*_, i.e. *μ*_*gt *_= *E*(*y*_*gti*_) for all *i*. A candidate gene expression profile of gene *g *is then given by inequalities between the components of ***μ***_*g *_= (*μ*_*g*1_, *μ*_*g*2_, ⋯, *μ*_*gT*_)^*T*^. In the following, we define some typical inequality profiles, and we drop the subscript *g *for simplicity.

*C*_0 _*profile *:

(1)

*C*_⊥ _*profile *:

(2)

where *μ*_*i*_⊥ *μ*_*j *_means that there is no defined inequality constraint between *μ*_*i *_and *μ*_*j*_.

*Monotone increasing profile *(*simple order*) :

(3)

(with at least one strict inequality). Similarly, a *monotone decreasing profile C*_↓ _is given by replacing ≤ by ≥ in (3).

*Up *-*down profile with maximum at i *(*umbrella order*) :

(4)

(with at least one strict inequality among *μ*_1 _≤ *μ*_2 _≤ ⋯ ≤ *μ*_*i *_and one among *μ*_*i *_≥ *μ*_*i*+1 _≥ ⋯ ≥ *μ*_*T*_). Genes satisfying this profile have mean expression values non-decreasing in time up to time point *i *and non-increasing thereafter. One may similarly define a *down-up profile C*_∨_.

*Cyclical profile with minima at *1, *j*, *and T and maxima at i and k *:

(5)

(with at least one strict inequality among each monotone sub-profile). Cyclical profiles may be important in relatively long time-course experiments where the mean expression value could oscillate.

*Incomplete inequality profiles *:

(6)

(with at least one strict inequality among each monotone sub-profile). Profiles (6) are useful when the investigator is unable to specify inequalities between certain means.

### Information-criterion based clustering using order-restricted maximum likelihood

Our procedure seeks to match a gene's true profile, estimated from the observed data, to one of a specified set of candidate profiles. Provided the relationship of a gene's mean expression levels at different time points is defined by a given candidate profile, we first obtain the order-restricted maximum likelihood estimates (MLE) of the gene's mean expression levels at all time points. Details for simple order and umbrella order constraints are given in the Methods section. A general discussion of order-restricted MLE can be found in [29]. Some specific examples can also be found in [1]. Peddada's method then carried out a bootstrap-based likelihood ratio test to decide a gene's best matched profile, which needs to repeat computing the order-restricted MLEs at least hundreds of times for each gene. We replace this very time-consuming bootstrap procedure by computing an information criterion function instead. The best matched profile is given by the one with the smallest information criterion function value. Information criterion functions, such as Akaike information criterion (AIC) [30] and Bayesian information criterion (BIC) [31], are widely used in selecting a model from a set of potential models. In our context, each candidate profile can be viewed as a potential model for a gene's expression pattern, and hence a properly defined information criterion function can be used to decide the gene's best matched profile. However, the widely used AIC and BIC are designed for models with a fixed number of parameters, whereas the inequality constraints do not really specify any parameters explicitly. Some information criteria for order-restricted inference have been proposed recently [32,33], but they only apply to simple order constraints (3). We have recently proposed a new order-restricted information criterion (ORIC) function for general inequality profiles, which we show to always select the correct profile when the sample size is large enough (unpublished manuscript). Suppose that there are *d *candidate profiles of interest. Let *l*(*λ*) denote the maximum log-likelihood under the *λ*-th candidate profile, and *ν*_1_(*λ*) and *ν*_2_(*λ*) are the number of ⊥ and {≥, ≤} specified in the profile, *λ *= 1, ..., *d*. Then, the ORIC function is

(7)

where *M *is the number of replicated arrays and

(8)

The ORIC function is similar to AIC and BIC in essence with *p*(*λ*) representing the model complexity. That is, the more the inequality constraints in a profile, the more complex it is as a model. And a profile with a smaller ORIC value is regarded as a better match to the gene's expression pattern. In the following, we describe our ORIC-based clustering (ORICC) algorithm (one-stage ORICC) and its computationally faster variant (two-stage ORICC).

#### One-stage ORICC

Step 1. Pre-specify a collection of candidate profiles, {*C*_1_, ..., *C*_*d*_}. To prevent genes with very little changes over time matched to these profiles, we also include *C*_0 _defined in (1) into the collection.

Step 2. Compute *p*(*λ*) in (8) for all candidate profiles.

Step 3. For gene *g*, compute the order-restricted MLE () of (*μ*_*g*1_, ⋯, *μ*_*gT*_) and the maximum log-likelihood *l*(*λ*) under each candidate profile *C*_*λ*_, *λ *= 0, 1, ⋯, *d*.

Step 4. For gene *g*, compute the information criterion function *ORIC*(*λ*) in (7) for all *λ *= 0, 1, ⋯, *d*. The best matched profile is then selected as that corresponds to  = arg min_0 ≤ *λ *≤ *d*_*ORIC*(*λ*), and gene *g *is assigned to the th cluster if  ≠ 0.

Step 5. Repeat Steps 3 and 4 for every gene.

Although our one-stage ORICC algorithm is hundreds of times faster than Peddada's method, performing Step 3 for all genes can still cost a lot of computational time even when only a moderate number of candidate profiles are considered because the number of genes is generally huge. This issue is more imminent for relatively longer time course microarray studies as more candidate profiles usually need to be considered. Next, we propose a computationally more efficient two-stage algorithm by adding a pre-screening stage.

#### Two-stage ORICC

Step 1. Pre-specify a collection of candidate profiles, {*C*_1_, ..., *C*_*d*_}. Here, we also add *C*_0 _and *C*_⊥ _defined in (1) and (2) into the collection for screening purpose.

Step 2. Compute *p*(*λ*) in (8) for all candidate profiles.

Step 3. For gene *g*, compute the order-restricted MLE () of (*μ*_*g*1_, ⋯, *μ*_*gT*_) and the maximum log-likelihood *l*(*λ*) under profiles *C*_0 _and *C*_⊥_.

Step 4. For gene *g*, compute *ORIC*(*λ*) in (7) for *λ *= 0, ⊥. Exclude gene *g *for further consideration if *ORIC*(0) <*ORIC*(⊥).

Step 5. Repeat Steps 3 and 4 for every gene. Denote the set of remained genes by *S*.

Step 6. Run Steps 3–5 in the one-stage ORICC algorithm for genes in *S *considering only {*C*_1_, ..., *C*_*d*_} as candidate profiles.

In the one-stage algorithm, the ORIC function is evaluated for every gene under every candidate profile, whereas the two-stage algorithm first screens out genes that show no significant changes over time by comparing between two profiles *C*_0 _and *C*_⊥_, and then applies the one-stage algorithm to a much smaller set of remained genes. As a result, the two stage algorithm is usually much faster and report tighter clusters with less genes in them.

#### Filtering genes with small expression levels

Some genes selected by the ORICC algorithm may have small mean expression levels at every time point. Such genes may not be of interest to some investigators. Peddada *et al*. [1] suggested a simple step to remove them. We include it here for completeness.

Let

(9)

where . Large values of *v*_*g *_indicate that the mean expression of gene *g *is high for at least one time point. Arrange the genes that selected by the ORICC algorithm in descending order of *v*_*g *_and retain the top *R *genes.

## Assessing the reliability of the oricc results

Microarray data are often noisy and hence it is important to assess the reliability of the clustering results. Among the recently developed methods for assessing clustering reliability [34-38], we adopt a general bootstrap framework proposed by Kerr and Churchill [36], in which the clustering procedure is first applied to the original data and then to a large number of bootstrap samples obtained from perturbing the original data. Since Peddada's method is computationally so costly, it is impossible to put it into this framework. By contrast, our ORICC method is computationally very efficient, and we can easily embed it into this general framework.

In time-course microarray studies, we can use the following analysis of variance (ANOVA) model to account for sources of variation in microarray data.

(10)

where *y*_*gti *_is the relative expression measurement from array *i*, time point *t*, and gene *g *on appropriate scale (typically the log scale). The terms *β*_*t*_, *γ*_*i *_and *ψ*_*ti *_account for all effects that are not gene-specific. We assume that the error terms *ε*_*gti *_are independent with mean 0 and variance  but do not make any other distributional assumption. The bootstrap assessment is then done as follows.

Step 1. Estimate model (10), which can be done straightforwardly in any statistics software, such as SAS [39] and R [40].

Step 2. Generate *B *bootstrap samples by



where a ^ over a term means the estimate from the original model fit in Step 1, and  are drawn with replacement from the studentized residuals of the original model fit.

Step 3. Repeat the ORICC algorithm for each bootstrap sample.

Now, the original clustering is accompanied by a collection of bootstrap clusterings, which can be regarded as a sample of clusterings that are close to the original clustering in space of all possible clusterings. When the level of noise in the original data is low, the bootstrap clusterings tend to be more like the original clustering. Then we can calculate a reliability measure for each gene by counting the proportion in the bootstrap clusterings it is attached to the same profile as in the original clustering. The larger the measure, the more reliable the gene's clustering membership.

### Simulation studies

In this section, we use Monte Carlo simulation to examine the performance of the ORICC method and compare it with other clustering methods for short time-course microarray data, including Peddada's method, STEM [2], and Wang's method [3].

The STEM algorithm works by assigning genes to a pre-defined set of model profiles that capture the potential distinct patterns that can be expected from the microarray experiment. Each gene is then assigned to the closest model profile in certain distance measure, e.g. correlation, and genes assigned to the same model profile consist a cluster. Significant profiles/clusters are next determined by hypotheses tests. As a result, genes in insignificant clusters are usually not reported. Wang's method represents each gene's temporal profile by a polynomial model and estimates the model using a Bayesian approach. A heuristic search strategy [23] is then applied to obtain clusters by repeatedly merging models to improve marginal likelihood.

All simulations were carried out on a workstation with a 2.30 GHz AMD Athlon(tm) 64 × 2 Dual Core 4400+ processor and a 2.00 GB memory. Peddada's method, Wang's method and the one-stage ORICC algorithm are implemented in R [40], whereas the STEM software is in JAVA written by its author.

#### Simulation 1

In the first simulation study, we consider ten inequality profiles (*C*_1_–*C*_10_) plus a flat pattern (*C*_0_) to represent a total number of eleven clusters. We set the number of time points as *T *= 6. These numbers are set to be similar to those in the real data set analyzed in the next section. The eleven profiles are specified as follows. For compactness, we drop *μ *∈ *R*^*T *^and the phrase 'with a strict inequality' when defining the profiles. True values of *μ *= (*μ*_1_, *μ*_2_, *μ*_3_, *μ*_4_, *μ*_5_, *μ*_6_) in each profile are also given.

C_0_: *μ*_1 _= *μ*_2 _= *μ*_3 _= *μ*_4 _= *μ*_5 _= *μ*_6_,    *μ *= (0,0,0,0,0,0);

C_1_: *μ*_1 _≥ *μ*_2 _≥ *μ*_3 _≥ *μ*_4 _≥ *μ*_5 _≥ *μ*_6_,    *μ *= (0,-0.5,-1,-1.5,-2,-2.5);

C_2_: *μ*_1 _≤ *μ*_2 _≤ *μ*_3 _≤ *μ*_4 _≤ *μ*_5 _≤ *μ*_6_,    *μ *= (0, 0.5, 1, 1.5, 2, 2.5)

C_3_: *μ*_1 _≤ *μ*_2 _≥ *μ*_3 _≥ *μ*_4 _≥ *μ*_5 _≥ *μ*_6_,    *μ *= (0, 0.5, 0, -0.5, -1, -1.5)

C_4_: *μ*_1 _≤ *μ*_2 _≤ *μ*_3 _≥ *μ*_4 _≥ *μ*_5 _≥ *μ*_6_,    *μ *= (0, 0.5, 1, 0.5, 0, -0.5)

C_5_: *μ*_1 _≤ *μ*_2 _≤ *μ*_3 _≤ *μ*_4 _≥ *μ*_5 _≥ *μ*_6_,    *μ *= (0, 0.5, 1, 1.5, 1, 0.5)

C_6_: *μ*_1 _≤ *μ*_2 _≤ *μ*_3 _≤ *μ*_4 _≤ *μ*_5 _≥ *μ*_6_,    *μ *= (0, 0.5, 1, 1.5, 2, 1.5)

C_7_: *μ*_1 _≥ *μ*_2 _≤ *μ*_3 _≤ *μ*_4 _≤ *μ*_5 _≤ *μ*_6_,    *μ *= (0, -0.5, 0, 0.5, 1, 1.5)

C_8_: *μ*_1 _≥ *μ*_2 _≥ *μ*_3 _≤ *μ*_4 _≤ *μ*_5 _≤ *μ*_6_,    *μ *= (0, -0.5, -1, -0.5, 0, 0.5)

C_9_: *μ*_1 _≥ *μ*_2 _≥ *μ*_3 _≥ *μ*_4 _≤ *μ*_5 _≤ *μ*_6_,    *μ *= (0, -0.5, -1, -1.5, -1, -0.5)

C_10_: *μ*_1 _≥ *μ*_2 _≥ *μ*_3 _≥ *μ*_4 _≥ *μ*_5 _≤ *μ*_6_,    *μ *= (0, -0.5, -1, -1.5, -2, -1.5)

**Figure 1:**
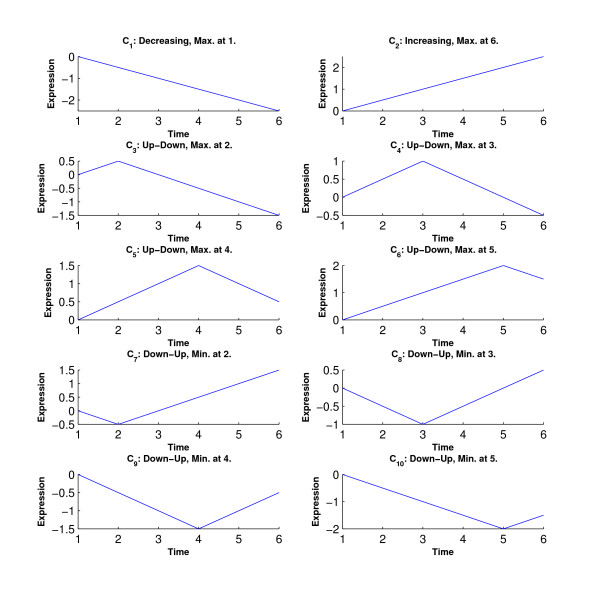
**Ten inequality profiles in Simulations 1 and 2**.

Figure 1 shows the inequality profiles *C*_1_–*C*_10_. We generated a data set with 200 genes from each profile.

At each time point *t*, we generated *M *replicates for each gene's expression level from normal distributions with means *μ*_*t *_and variance *σ*^2^. To assess the effect of the data variability and replicates on the clustering results, we varied the variance *σ*^2 ^from 0.2 to 1.2 (by an incremental of 0.2) and the number of replicates *M *from 2 to 10.

Next, we clustered the simulated data using Peddada's method and the one-stage ORICC algorithm, considering ten candidate inequality profiles *C*_1_–*C*_10_. For Peddada's method, we set the number of bootstrap replications as 200 and the significance level of the bootstrap based test as 0.025. Peddada *et al*. originally proposed to use significance level 0.0025, and we have observed that a large number of "non-flat" genes will be clustered to *C*_0 _using this choice. Meanwhile, using the common significance level of 0.05 tends to cluster many genes from *C*_0 _to other "non-flat" profiles. Using significance level of 0.025 offers a good compromise between the two kinds of false clustering.

Let *γ*_*i *_denote the number of genes with true profile *C*_*i *_and correctly clustered to profile *C*_*i*_, *i *= 0, 1, ..., 10. The *overall error rate *and the *false positive rate *are then given by /(11 × 200) and 1 -*γ*_0 _= 200, respectively. Let  denote the number of genes with true profile *C*_*i*_, *i *= 1, ..., 10 and clustered to profile *C*_0_. The *false negative rate *is then given by /(10 × 200).

**Figure 2:**
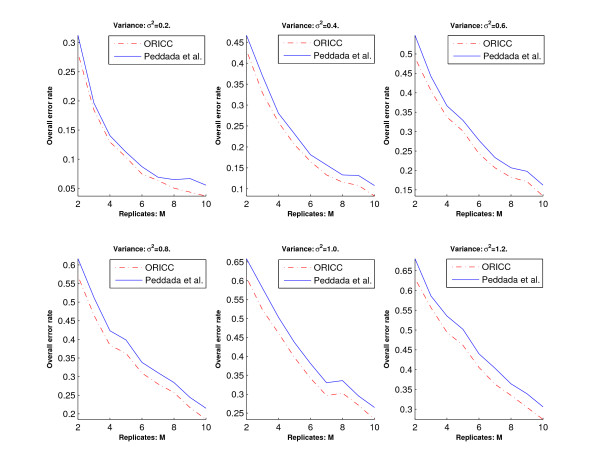
**Simulation 1: The overall error rate of Peddada's method and the one-stage ORICC algorithm**. The horizontal axis represents the number of replicates, and the vertical axis represents the overall error rate. Dashed lines are for the one-stage ORICC algorithm, and solid lines are for Peddada's method.

**Figure 3:**
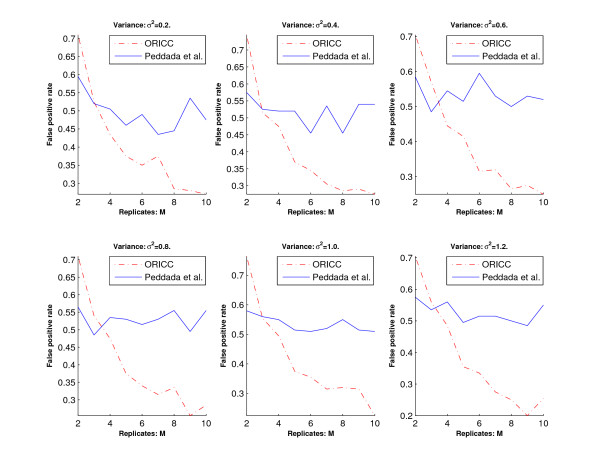
**Simulation 1: The false positive rate of Peddada's method and the one-stage ORICC algorithm**. The horizontal axis represents the number of replicates, and the vertical axis represents false positive rate. Dashed lines are for the one-stage ORICC algorithm, and solid lines are for Peddada's method.

**Figure 4:**
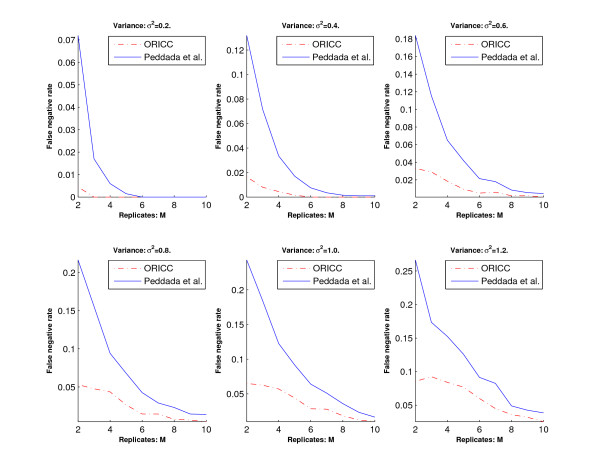
**Simulation 1: The false negative rate for Peddada's method and the one-stage ORICC algorithm**. The horizontal axis represents the number of replicates, and the vertical axis represents the false negative rate. Dashed lines are for the one-stage ORICC algorithm, and solid lines are for Peddada's method.

We then use the overall error rate, the false positive rate and the false negative rate to evaluate the accuracy of the two algorithms. Simulation results are summarized in Figures 2, 3 and 4. Figure 2 and Figure 4 show that the overall error rate and the false negative rate of the one-stage ORICC algorithm are always better than those of Peddada's method. Figure 3 shows that the false positive rate of the one-stage ORICC algorithm is better than that of Peddada's method in most cases. The one-stage ORICC algorithm not only provides good clustering accuracy but also is much faster than Peddada's method. For example, when *σ*^2 ^= 1 and *M *= 5, the run time for Peddada's method and one-stage ORICC algorithm is 2979.29 seconds versus 25.55 seconds.

#### Simulation 2

In the second simulation study, we consider the same set of inequality profiles and simulate the data in the same way as in Simulation 1, but we fix the number of replicates *M *to be 5. To study the effect of the true cluster size to the clustering accuracy, we consider different cluster sizes, 50, 100, 150 and 200. Meanwhile, we also vary the variance *σ*^2 ^from 0.2 to 3.0 with an incremental of 0.4. Then we cluster the simulated data set using methods including Peddada's method, Wang's method, STEM and the one-stage ORICC algorithm.

**Figure 5:**
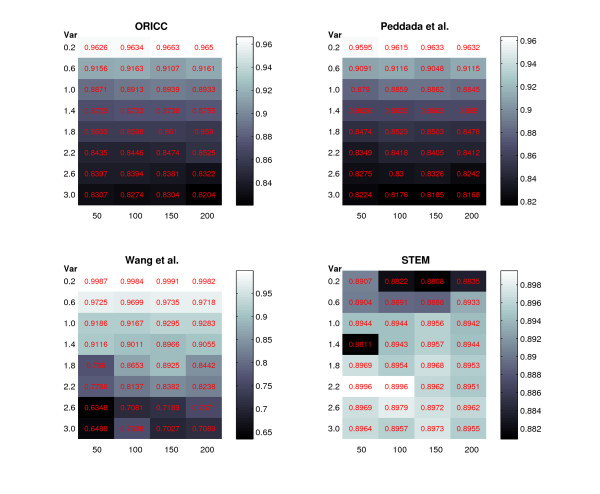
**Simulation 2: Clustering precision of four different methods**. The numbers in the cells are Rand's *C *statistics. The intensity of the color corresponds to the magnitude of the number as shown by the legend. The horizontal axis represents the cluster size, and the vertical axis represents the data variance *σ*^2^.

For Peddada's method and the one-stage ORICC algorithm, we consider eleven candidate inequality profiles *C*_0_–*C*_10_. For Wang's method, we set the prior hyper-parameters (*α*_1_, *α*_2_) in the gamma prior distribution *Gamma*(*α*_1_, *α*_2_) as (2, 2). For STEM, we assume 50 possible profiles and use the recommended default settings in the package. To be consistent, we did not filter out any genes in any of these analyses. Then we use Rand's *C *statistic [41] to evaluate the similarity between the true cluster assignment and the clustering results of different methods. Rand's *C *statistic is defined as follows. Given a pair of clusterings *C *and *C' *of the same *N *objects, arbitrarily number the clusters in each clustering and let *n*_*ij *_be the number of objects simultaneously in the *i*th cluster of *C *and the *j*th cluster of *C'*. Then, Rand's *C *statistic is given by



which denotes the proportion of pairs of objects that are assigned consistently in the two clusterings. Figure 5 gives Rand's *C *statistic from different clustering methods for different *σ*^2 ^and cluster sizes. It shows that the precision of all methods is decreasing for increasing variance, and the cluster size has no obvious effect on the clustering precision for Peddada's method, STEM and the one-stage ORICC algorithm, but has an increasing effect for Wang's method. This comparison shows an interesting pattern. For larger *σ*^2^, STEM performs the best, Wang's method the worst, and Peddada's method and the one-stage ORICC are in between. For smaller *σ*^2^, the result is reversed with STEM being the worst, Wang's method the best, and Peddada's method and the one-stage ORICC still in between. When the cluster size is relatively small and *σ*^2 ^is large, Wang's method can have quite low precision under 70%. Overall, the one-stage ORICC algorithm is consistently more accurate than Peddada's method by a slight margin, and provides good precision under all scenarios. The performance of STEM is also very stable, but tends to underperform when the data are less noisy, i.e., *σ*^2 ^is small.

**Figure 6:**
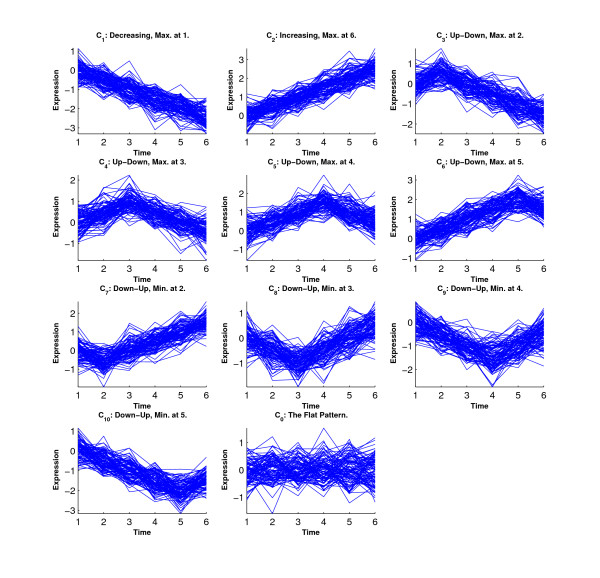
**Simulation 2: The simulated eleven clusters when *M *= 5, *σ *= 1 and cluster size = 100**.

**Figure 7:**
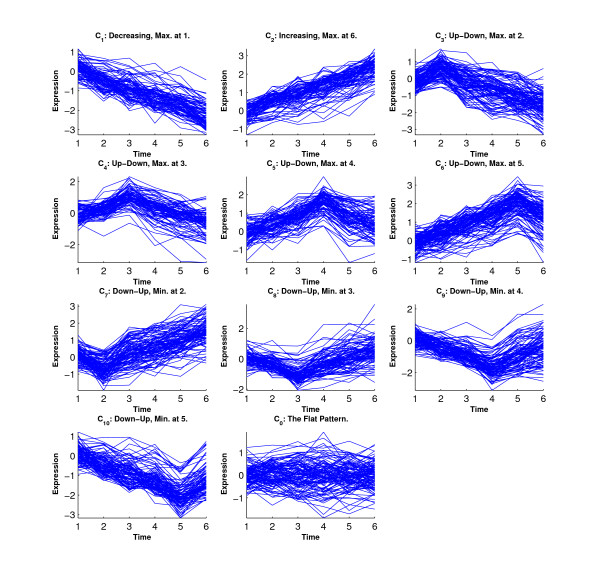
**Simulation 2: Temporal profiles for clusters from the ORICC analysis**.

**Figure 8:**
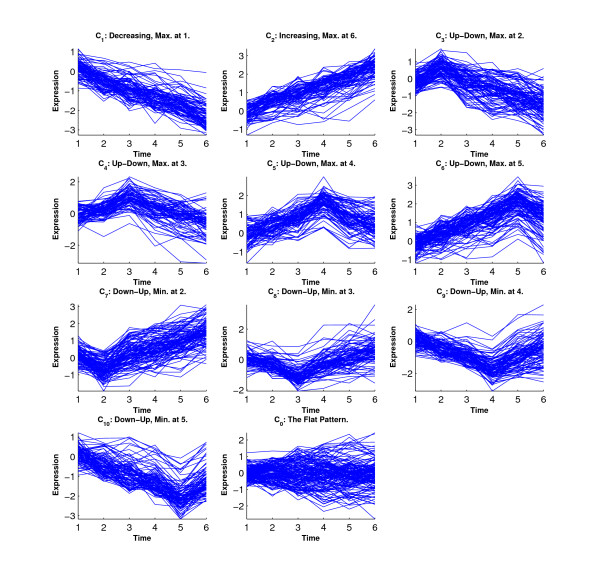
**Simulation 2: Temporal profiles for clusters from Peddada's method**.

**Figure 9:**
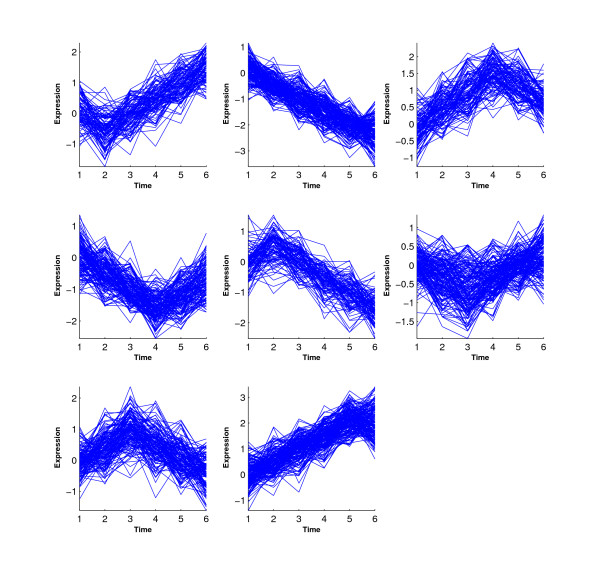
**Simulation 2: Temporal profiles for clusters from Wang's method**.

**Figure 10:**
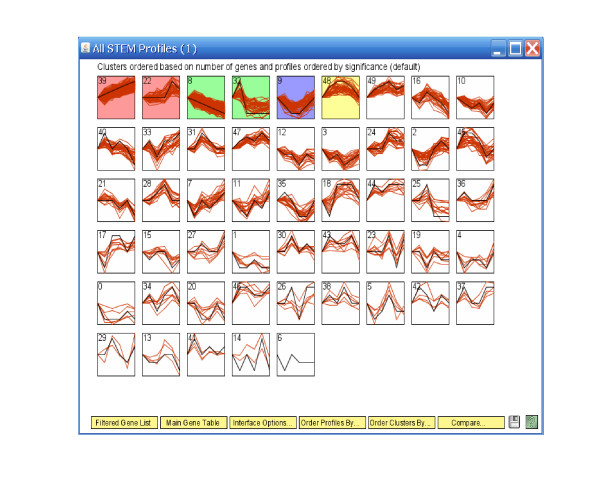
**Simulation 2: Temporal profiles for clusters from the STEM analysis**. The black curves are pre-specified model profiles.

Figure 6 shows the simulated eleven clusters when *M *= 5, *σ*^2 ^= 1 and the cluster size is 100. Figures 7, 8, 9 and 10 show the resulted clusters from the four different methods, respectively. ORICC and Peddada's method give similar clustering results that well match the true pattern. While the true number of clusters is eleven, STEM identifies six significant clusters and Wang's method keeps eight clusters. In particular, Wang's method did not cluster genes from the flat profile *C*_0 _into one cluster but assign them into different clusters.

In this simulation, Peddada's method, Wang's method and the one-stage ORICC method are implemented in R, whereas the STEM software is in JAVA written by its author. So, we can only compare the computational efficiency of the first three methods and the one-stage ORICC method is much faster than the other two. For example, when *σ*^2 ^= 3 and the cluster size is 200, the run time for Peddada's method, Wang's method and the one-stage ORICC algorithm is 3073.37 seconds, 10303.9 seconds and 24.72 seconds, respectively.

### Simulation 3

In the third simulation, we examine the robustness of the ORICC algorithm. We consider eleven inequality profiles (*C*_0_–*C*_10_) plus a cyclical profile (*C*_∧∧_) to represent a total number of twelve clusters. We set the number of time points as *T *= 6. The eleven inequality profiles *C*_0_–*C*_10 _and the true values of *μ *= (*μ*_1_, *μ*_2_, *μ*_3_, *μ*_4_, *μ*_5_, *μ*_6_) in each profile are the same as in Simulation 1. The cyclical profile *C*_∧∧ _and the true value of *μ *= (*μ*_1_, *μ*_2_, *μ*_3_, *μ*_4_, *μ*_5_, *μ*_6_) in *C*_∧∧ _are given as follows:



We generate a data set with 200 genes from each profile of *C*_0_–*C*_10 _and 200 × *r *genes from cyclical profile *C*_∧∧_. At each time point *t*, we generated 5 replicates for each gene's expression level from normal distributions with means *μ*_*t *_and variance *σ*^2^. To study the robustness of the one-stage ORICC algorithm, we consider different cluster sizes, 200 × *r*, *r *= 1, 2, ⋯, 10, for the cyclical profile *C*_∧∧_. Meanwhile, we also vary the variance *σ*^2 ^from 0.2 to 3.0 with an incremental of 0.4. Then we cluster the simulated data set using the one-stage ORICC algorithm. For the one-stage ORICC algorithm, we consider eleven candidate inequality profiles *C*_0 _– *C*_10_. Note that the cyclical profile *C*_∧∧ _is not included in the candidate profiles.

**Figure 11:**
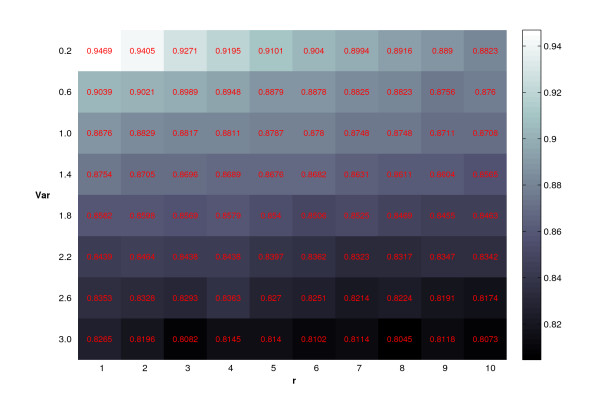
**Simulation 3: Clustering precision of the one-stage ORICC algorithm**. Numbers in the cells are Rand's *C *statistics. The intensity of the color corresponds to the magnitude of the number as shown by the legend. The horizontal axis represents the cluster size of the cyclical profile *C*_∧∧_, and the vertical axis represents the data variance *σ*^2^.

Then we use Rand's *C *statistic to evaluate the similarity between the true cluster assignment and the clustering results from ORICC. Figure 11 gives Rand's *C *statistic from the one-stage ORICC algorithm for different *σ*^2 ^and cluster sizes of the cyclical profile *C*_∧∧_. It shows that the precision of the one-stage ORICC algorithm as measured by Rand's *C *statistic increases for decreasing variance, and decreases when increasing the cluster size of the cyclical profile *C*_∧∧_. The cluster precision is however always greater than 80%, thus suggesting that the one-stage ORICC algorithm is very stable.

### Simulation 4

In the fourth simulation, we further examine the robustness of the ORICC algorithm. We consider the scenario where a true profile is not explicitly included in the candidate profiles but maybe viewed as a special case of a more flexible candidate profile. Let the true inequality profile be



**Figure 12:**
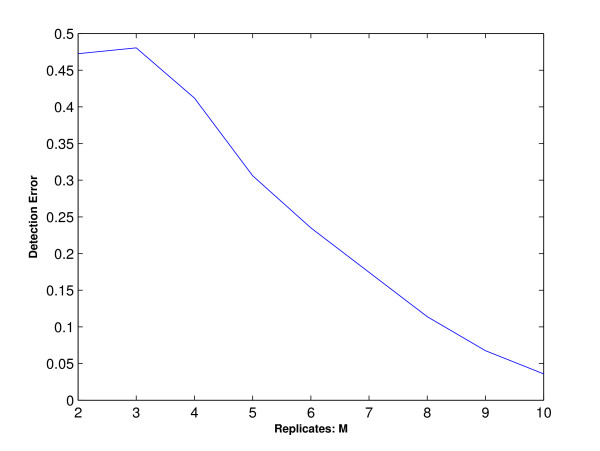
**Simulation 4: Detection error rate**. This figure plots the detection error rate of the one-stage ORICC algorithm for a true profile that is not explicitly specified in the candidate profiles.

We generate a data set containing 2000 genes from this profile. At each time point *t*, we generate *M *replicates for each gene's expression level from normal distributions with means *μ*_*t *_and variance 0.5. Then, we consider candidate profiles being *C*_1_, *C*_2_, *C*_4 _and *C*_9 _plus the profile *C*_⊥ _and cluster the simulated data using the one-stage ORICC algorithm. Note that the set of candidate profiles does not contain the true one *C*_∧∧_, but *C*_∧∧ _may be viewed as a special case of *C*_⊥_. Let *γ*_⊥ _denote the proportion of genes clustered to the profile *C*_⊥_, and define the detection error as 1 - *γ*_⊥_. The simulation results are summarized in Figure 12. It shows that the detection error decreases rapidly as the number of replicates M increases. With 5 replicates, the detection error is below 30%, and with 10 replicates, it goes down to below 5%. This indicates that it is quite safe to apply the ORICC algorithm even if some true profile is missing from the candidate profiles but a more comprehensive profile is considered.

### Application to breast cancer cell line data

Next, we apply the ORICC algorithm to log-transformed relative expression data from a breast cancer cell line microarray study in [28]. The same data set was also analyzed by Peddada *et al*. [1]. The experiment was done as follows. First, the MCF-7 breast cancer cell line was treated with 17*β*-estradiol or ethanol (vehicle control). Then, samples were harvested at 1, 4, 12, 24, 36 and 48 hours after treatment. At each time point, *M *= 8 replicate arrays were prepared with each array consisting of *G *= 1901 genes. Similar as in [1], we assumed for each gene that the variance of the log relative expression was homoscedastic over time, and consider the following 10 candidate profiles for clustering: monotone decreasing, *C*_1_; monotone increasing, *C*_2_; four up-down profiles with maxima at 4, 12, 24, 36 hours, *C*_3 _– *C*_6_, respectively; and 4 down-up profiles with minima at 4, 12, 24, 36 hours, *C*_7 _– *C*_10_, respectively. Genes matched to these profiles will be regarded estrogen responsive.

**Figure 13:**
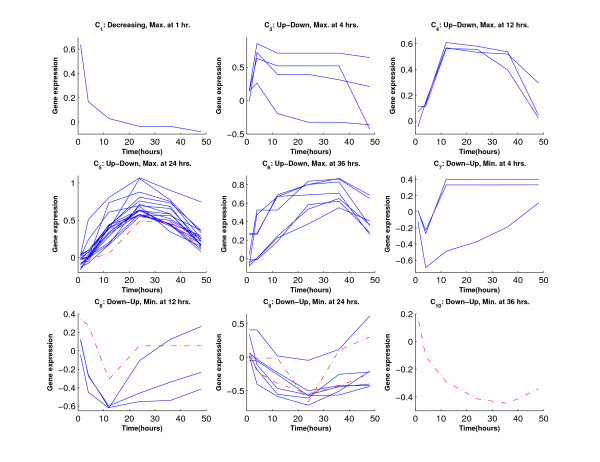
**Breast cancer cell line data: Estimated profiles of the top 50 genes**. Curves are given by the order-restricted MLE of mean log expression ratios. Dashed lines indicate newly identified genes.

The original analysis in [28] used a simple confidence interval approach [42], and identified 105 genes that demonstrated estrogen responsive expression. From the one-stage ORICC analysis, we finally had 981 genes in the 10 clusters, with 68 in *C*_1_, 24 in *C*_2_, 76 in *C*_3_, 44 in *C*_4_, 97 in *C*_5_, 72 in *C*_6_, 35 in *C*_7_, 98 in *C*_8_, 409 in *C*_9_, and 58 in *C*_10_. Due to limitation of space, we only present the top 50 genes ranked by the filtering criterion in (9) (Additional file 1). The last column in Additional file 1 indicates whether the gene was previously identified in [28]. Clustering reliability for each gene is also attached. Note that these 50 genes come from only nine of the 10 clusters, with none from *C*_2_. Figure 13 presents the estimated profiles of these 50 genes from the order-restricted MLE. Of the 105 genes identified in [28], 44 are among our top 50, 82 are among our top 100, 94 were among our top 150, and 101 were among our top 200. Most of the 44 genes in the top 50 selected in common are involved in cell cycle progression and DNA replication reflecting the known sensitivity of MCF-7 cells to estrogen. Among the six genes identified by our ORICC algorithm but not in [28] (denoted by dashed lines in Figure 13), two have quite high clustering reliability. The methylmalonyl Coenzyme A mutase (Clone ID 35468 in *C*_9_) has reliability 0.9967, and the deoxythymidylate kinase (Clone ID 489092 in *C*_5_) has reliability 0.7800. Both genes are known specific to the metabolic process, and hence are very likely responsive to the metabolism of estrogen when overdosed estrogen are supplied to the cell. For example, the methylmalonyl Coenzyme A mutase could be involved in the breakdown of estradiol into smaller metabolic fragments. However, this gene was not reported in the top 50 list by Peddada *et al*. [1]. Estimated profiles in Figure 13 suggests this gene matches very well with the candidate profile *C*_9_. An interesting phenomenon about the deoxythymidylate kinase is that this gene actually corresponds to two spots on the microarray chips (Clone IDs 489092 and 248008). The original analysis in [28] was only able to identify one of them (Clone ID 248008) but not the other, whereas our method identifies both in the same cluster with high clustering reliability.

In [1], Peddada *et al*. reported that 39 genes in the top 50 were in common with the 105 genes selected in [28]. Our ORICC result has a good overlap with Peddada's result, with 32 genes in our top 50 also in the top 50 list reported in [1]. Peddada *et al*. discussed in details several newly identified genes in their top 50, such as replication factors C4 and C5. Our ORICC analysis also cluster these genes into the same clusters, but they are not in our top 50 list due to relatively low clustering reliability, with replication factor C4 ranked 75 and replication factor C5 ranked 148.

**Figure 14:**
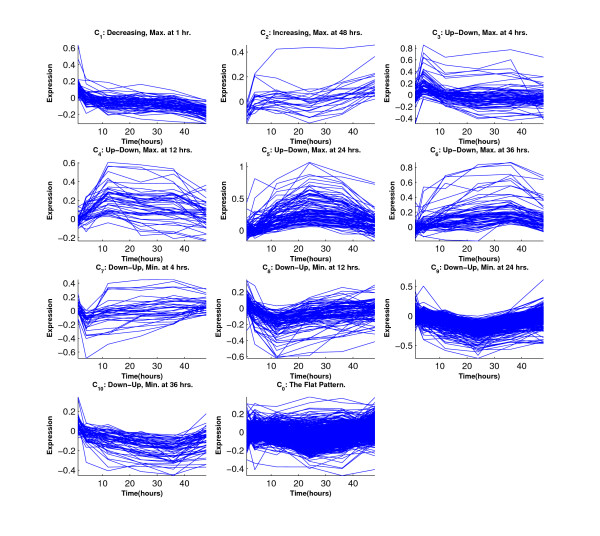
**Breast cancer cell line data: Temporal profiles of clusters from the ORICC analysis**. Curves are given by connecting the observed log expression ratios at different time points.

**Figure 15:**
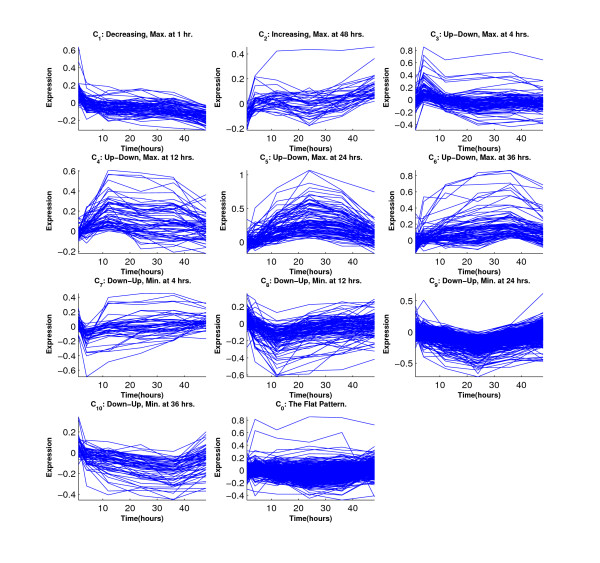
**Breast cancer cell line data: Temporal profiles of clusters from Peddada's method**. Curves are given by connecting the observed log expression ratios at different time points.

**Figure 16:**
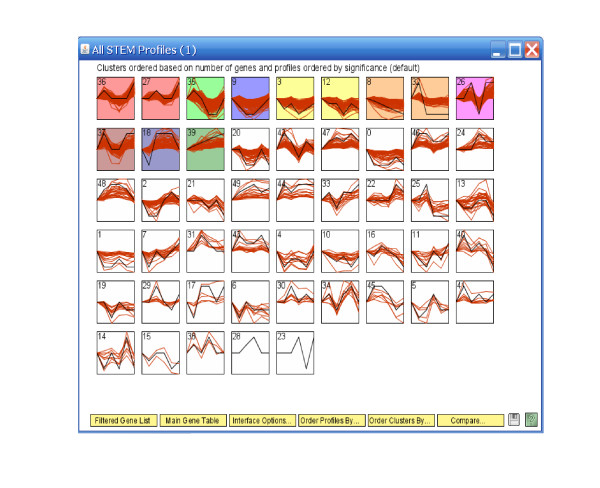
**Breast cancer cell line data: Temporal profiles of clusters from the STEM analysis**. Curves are given by connecting the observed log expression ratios at different time points. And the black curves are pre-specified model profiles.

**Figure 17:**
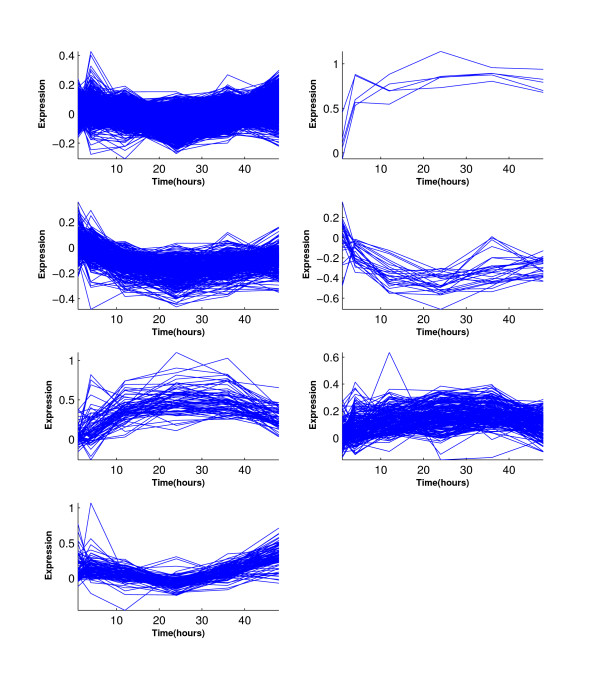
**Breast cancer cell line data: Temporal profiles of clusters from Wang's method**. Curves are given by connecting the observed log expression ratios at different time points.

We further applied STEM and Wang's method on the breast cancer cell line data. Table 1 reports Rand's C statistics among results from four clustering methods. It shows that the three profile matching algorithms have results more alike each other, while the unsupervised Wang's method is less similar to the rest. This observation can also be seen in the temporal profiles of the clusters given by four different methods (Figures 14, 15, 16 and 17). While ORICC and Peddada's method give ten clusters plus the 'flat' cluster, Wang's method identifies seven clusters, and STEM reports twelve significant clusters.

**Table 1:** Rand's C statistics among four clustering methods in the breast cancer cell line example.

	ORICC	Peddada	Wang	STEM
ORICC	1.0000	0.7767	0.6313	0.7142
Peddada	0.7767	1.0000	0.5948	0.7694
Wang	0.6313	0.5948	1.0000	0.6025
STEM	0.7142	0.7694	0.6025	1.0000

A larger value indicates more overlap between two clustering results.

## Discussion

In time-course microarray experiments, the ability to exploit the temporal ordering information may be especially valuable because genes whose expression levels change over time may be involved in the same cellular process or belong to the same regulatory pathway. Making use of the valuable ordering information can improve inference. Our proposed ORICC algorithm utilizes the temporal ordering information in clustering time-course microarray data using order-restricted maximum likelihood, while most existing clustering methods either can not incorporate the temporal information or require long time series to perform reliable nonparametric smoothing, *e.g*. spline smoothing, though most time-course microarray data are short time series. In our method, the temporal ordering information is exploited through a set of pre-defined candidate expression profiles given by inequality constraints among the mean expression levels at different time points. By viewing each candidate profile as a potential model for the data and let each profile represent a cluster, we transform the clustering problem into a model selection problem. Using an ORIC function, we decide the best matched profile for each gene and hence determine the gene's clustering membership. Peddada *et al*. [1] instead performed a likelihood based test to decide the best matched profile. However, a bootstrap procedure is needed to decide the threshold value on the test statistic, which makes Peddada's method computationally very intensive.

In many situations, field researchers can have good ideas on defining the inequality profiles. For example, when studying the gene expression patterns for disease onset. It is easy to postulate that gene expressions tend to go up before the disease onset and then go down after certain treatment is given. So, the inequality constraints allow an easy adoption of a prior knowledge into the analysis, whereas existing methods usually can not take such information into consideration. In addition, when inequality constraints are given, the order-restricted MLE has some optimal properties and universally dominates the unrestricted MLE [43]. Moreover, the candidate profiles are defined only based on ranks instead of the numerical value of the mean expression levels, hence our model specification is robust to small perturbation in the data. This feature is especially valuable in microarray studies since it is well known that microarray data are quite noisy. Furthermore, the rank-based specification is often closer to the real meaning of 'coexpression' that refers to two genes' expression levels changing in the same direction instead of with the same magnitude.

In this paper, we present our algorithm under the context of clustering time-course microarray data. Actually, it can be applied to data from any experiment with ordered treatment or conditions, such as dose-response microarray experiments where the dose levels provide the ordering.

Our current ORICC algorithm is based on order-restricted MLE for gene expressions with a constant variance through time. It can be generalized to handle situations where the variances change or are subject to order restrictions themselves. In such situations, the estimation of mean expression levels outlined in this paper can be modified according to the approach in [44]. However, it remains a subject for future investigation to modify the definition of the model complexity *p*(*λ*) (8) accordingly.

## Conclusion

We developed a new clustering algorithm, ORICC, for short time-course microarray data, by taking a model selection approach in order-restricted statistical inference. Our method clusters genes into clusters represented by candidate profiles defined through inequalities among mean expression levels at different time points. A newly proposed information criterion function is used to determined the cluster assignment. Compared with a previous clustering method by Peddada *et al*. [1] that also uses order-restricted inference, our method is computationally much more efficient and provides an assessment of clustering reliability. Simulation studies indicate that the ORICC algorithm possesses good clustering accuracy when a moderate number of replicate arrays are available, and competes well with other existing clustering methods, such as [2] and [3]. Real data applications also indicate that our method can identify interesting genes that some correlation-based methods have failed to identify.

## Methods

### Order-restricted maximum likelihood estimation

Here, we briefly present the order-restricted MLE under simple order (3) and umbrella order constraints (4), which are needed for ORICC analysis in our simulation and real data example. For more general results, we refer to [29,45]. Suppose that *y*_*ti*_'s independent observations from normal distributions with unknown means *μ*_*t *_and variances *v*_*t *_for *t *= 1, ⋯, *T *and *i *= 1, ⋯, *n*_*t*_. Then the data log-likelihood is

(11)

Where , ***μ ***= (*μ*_1_,⋯, *μ*_*T*_)^*T *^and **v **= (*v*_1_, ⋯, *v*_T_)^*T*^. Let *w*_*t*_ = *n*_*t*_/*v*_*t*_,  and . When assuming the variances **v **are known, we have the order-restricted MLE of ***μ ***as

(1) *μ*_1 _≤ *μ*_2 _≤ ⋯ ≤ *μ*_*T*_:



(2) *μ*_1 _≥ *μ*_2 _≥ ⋯ ≥ *μ*_*T*_:



(3) *μ*_1 _≤ ⋯ ≤ *μ*_*h *_≥ ⋯ ≥ *μ*_*T*_:



(4) *μ*_1 _≥ ⋯ ≥ *μ*_*h *_≤ ⋯ ≤ *μ*_*T*_:



The maximum log-likelihood under the *λ*-th candidate profile is then obtained by plugging in the corresponding order-restricted MLE into (11).

If the variances **v **are unknown, we need to impose the assumption *v*_1 _= *v*_2 _= ⋯ = *v*_*n *_= *v*. In microarray data, this assumption is reasonable after the data are properly normalized. Now, the data log-likelihood is

(12)

Under this situation, the order-restricted MLE of ***μ ***can be obtained similarly as in the known variance case by letting *w*_*t *_= *n*_*t *_instead of *w*_*t *_= *n*_*t*_/*v*_*t*_. And the MLE of *v *is then



Again, the maximum log-likelihood for the *λ*-th candidate profile is then obtained by plugging in the corresponding order-restricted MLE into (12).

## Availability and requirements

We have implemented ORICC in an R program, which can be downloaded from .

## Authors' contributions

NL, NS and BZ had the initial idea and initiated the study. NL and TL conducted the data analyses, created all tables and figures, and prepared the manuscript under the supervision of BZ. All authors read and approved the final manuscript.

## Supplementary Material

Additional File 1 **Gene clusters from the ORICC analysis of the breast cancer cell line data**. This table presents gene clusters given by the ORICC algorithm using ten candidate inequality profiles for the breast cancer cell line microarray data in [28]. The fourth column gives the rank of the gene according to the filtering criterion (9), and numbers in the parentheses are clustering reliability computed based on 300 bootstrap samples. The last column shows whether the gene was identified by the original analysis in [28]. Click here for file
