# Supplementary material for: Information criterion-based clustering with order-restricted candidate profiles in short time-course microarray experiments
Source: BMC Bioinformatics. 2009 May 15;10:146. doi: 10.1186/1471-2105-10-146 (PMC2696449; doi:10.1186/1471-2105-10-146)
Supplement: Additional File 1 — Gene clusters from the ORICC analysis of the breast cancer cell line data. This table presents gene clusters given by the ORICC algorithm using ten candidate inequality profiles for the breast cancer cell line microarray data in [28]. The fourth column gives the rank of the gene according to the filtering criterion (9), and numbers in the parentheses are clustering reliability computed based on 300 bootstrap samples. The last column shows whether the gene was identified by the original analysis in [28]. [file 1471-2105-10-146-S1.pdf]

TABLE 1. Gene clusters from the ORICC analysis

Clone ID	Gene name	Function category	Top (Reliability)	Previously identified
C1				
417226	v-myc viral oncogene homolog	Transcription/Chromatin Structure	22 (0.7067)	Yes
C3				
489175	acid phosphatase1,soluble	Cellular Signaling	2 (0.7400)	Yes
428733	protein kinase C,delta	Cellular Signaling	25 (0.8667)	Yes
248613	v-myb viral oncogene homolog	Transcription/Chromatin Structure	39 (0.7333)	Yes
362059	alpha3	Extracellular Matrix/Cell Structure	40 (0.7533)	Yes
C4				
591098	polymerase,delta1,catalytic subunit	DNA replication/repair	23 (0.6167)	Yes
344109	proliferating cell nuclear antigen	DNA replication/repair	34 (0.6133)	Yes
321207	polymerase,epsilon	DNA replication/repair	41 (0.4600)	Yes
C5				
198205	v-myb viral oncogene homolog-like2	Transcription/Chromatin Structure	1 (0.9200)	Yes
339075	karyopherin alpha2	Protein degradation/synthesis/targeting	3 (1.0000)	Yes
52713	vitronectin	Extracellular Matrix/Cell Structure	4 (0.9033)	Yes
136609	v-myb viral oncogene homolog-like1	Transcription/Chromatin Structure	5 (0.9800)	Yes
248008	deoxythymidylate kinase	Cell cycle	10 (0.9100)	Yes
415639	serine/threonine kinase15	Cell cycle	11 (0.9700)	Yes
563809	CDC20 cell division cycle20 homolog	Cell cycle	13 (0.9933)	Yes
129140	MAD2 mitotic arrest deficient-like1	Cell cycle	14 (0.9467)	Yes
49950	flap structure-specific endonuclease1	DNA replication/repair	16 (0.7300)	Yes
32811	polymyositis/scleroderma autoantigen1	Cell cycle	18 (0.7933)	Yes
198233	thyroid hormone receptor interactor13	Transcription/Chromatin Structure	27 (0.7900)	Yes
359119	CDC28 protein kinase2	Cell cycle	28 (0.9333)	Yes
200573	ESTs	Unknown	30 (0.9100)	Yes
359465	dihydrofolate reductase	DNA replication/repair	31 (0.5967)	Yes
488059	tubulin, gamma1	Cell cycle	32 (0.8800)	Yes
417703	serine/threonine kinase12	Cell cycle	33 (0.9133)	Yes
489092	deoxythymidylate kinase	Cell cycle	36 (0.7800)	No
293274	cyclin-dependent kinase inhibitor3	Cell cycle	44 (0.5433)	No
1144761	low density lipoprotein receptor	Miscellaneous	50 (0.4433)	Yes
C6				
242182	protein kinase inhibitor beta	Cellular Signaling	6 (0.8500)	Yes
150163	neuropeptide Y receptor Y1	Cellular Signaling	12 (0.6933)	Yes
278533	cytochrome c oxidase subunit VIc	Miscellaneous	19 (0.9167)	Yes
509614	high-mobility group protein1	Transcription/Chromatin Structure	20 (0.8200)	Yes
530696	MCM7 minichromosome maintenance deficient7	DNA replication/repair	24 (0.7333)	Yes
510595	lactate dehydrogenase A	Miscellaneous	35 (0.8067)	Yes
417125	7-dehydrocholesterol reductase	Miscellaneous	47 (0.3300)	Yes
C7				
487407	insulin induced gene1	Miscellaneous	15 (0.9000)	Yes
297392	metallothionein 1L	Miscellaneous	17 (0.9867)	Yes
356890	hyaluronoglucosaminidase1	Extracellular Matrix/Cell Structure	42 (0.6800)	Yes

TABLE 1. continued.

Clone ID	Gene name	Function category	Reliability	Previously identified
C8				
484963	metallothionein 2A	Miscellaneous	8 (0.9867)	Yes
485875	EGF-containing fibulin-like extracellular matrix protein1	Extracellular Matrix/Cell Structure	21 (0.7867)	Yes
361381	myeloid cell leukemia sequence1	Apoptosis	37 (0.8200)	Yes
47994	pyruvate kinase, muscle	Unknown	49 (0.4433)	No
C9				
162479	E74-like factor3	Transcription/Chromatin Structure	7 (0.8933)	Yes
35468	methylmalonyl Coenzyme A mutase	Unknown	9 (0.9967)	No
430235	H2B histone family, member Q	Transcription/Chromatin Structure	26 (0.9200)	Yes
359191	protein kinase H11	Cellular Signaling	29 (0.8600)	Yes
588492	Homo sapiens cDNA FLJ	Unknown	38 (0.7300)	Yes
161566	NAD(P)H dehydrogenase, quinone1	Miscellaneous	43 (0.4600)	Yes
268652	cyclin-dependent kinase inhibitor 1A	Cell Cycle	46 (0.5367)	No
180789	low density lipoprotein-related protein1	Protein degradation/synthesis/targeting	48 (0.4900)	Yes
C10				
365147	v-erb-b2 viral oncogene homolog2	Cellular Signaling	45 (0.4800)	No
